# SLEEP AND DEPRESSIVE SYMPTOMS IN COMMUNITY-DWELLING OLDER ADULTS: FINDINGS FROM AMBULATORY SLEEP EEG

**DOI:** 10.1093/geroni/igad104.1285

**Published:** 2023-12-21

**Authors:** Jing Huang, Adam Spira, Miranda McPhillips, Russell Calderon, Mengchi Li, Yeabsira Tufa, Junxin LI

**Affiliations:** Johns Hopkins University, Baltimore, Maryland, United States; Johns Hopkins Bloomberg School of Public Health, Baltimore, Maryland, United States; The University of Pennsylvania, Philadelphia, Pennsylvania, United States; Johns Hopkins University, Baltimore, Maryland, United States; Johns Hopkins University, Baltimore, Maryland, United States; Johns Hopkins University, Baltimore, Maryland, United States; Johns Hopkins University, Baltimore, Maryland, United States

## Abstract

There is limited and inconsistent evidence on the association between electroencephalography (EEG) measured sleep and depressive symptoms among community-dwelling older adults. This study investigated the cross-sectional association between EEG-measured sleep and depressive symptoms in 72 community-dwelling older adults without dementia [Age> 65 years; Montreal Cognitive Assessment (MoCA)>17], using baseline data from a randomized clinical trial (NCT03959202). Sleep and depressive symptoms were measured using two-night in-home sleep EEG (Sleep Profiler™) and the Geriatric Depression Scale (GDS-15). Multiple linear regression analyses were conducted with each sleep parameter as the primary predictor and GDS score as the outcome; models were adjusted for age, sex, race, education, marital status, chronic conditions, and MoCA score. Several sleep variables were associated with depressive symptoms (GDS score), including a higher percentage of sleep stage N1 (b = 0.11, 95% confidence interval [CI]: 0.02 – 0.20) and N2 (b = 0.04, 95% CI: 0.00 – 0.08), a lower percentage of N3 sleep (b = -0.04, 95%CI: -0.08 – -0.01), greater wake after sleep onset [b = 0.01, 95%CI: 0.00 – 0.02], and more awakenings ≥ 90s/hour (b = 0.87, 95%CI: 0.21–1.53). Neither total sleep time nor sleep efficiency were associated with GDS score. In conclusion, we found that more lighter (stage N1, N2) sleep, less deep (N3) sleep, and more fragmented sleep, measured by EEG, were associated with more depressive symptoms among community-dwelling older adults without dementia. Sleep architecture and fragmentation may play more important roles than sleep duration in relation to depression.

